# Communications Respecting the Epidemic Cholera at St. Petersburg, &c.

**Published:** 1837-04

**Authors:** 


ART. X.—1. *Mittheilungen ueber die Cholera Epidemie zu St. Petersburg, im Sommer* 1831*; von Praktischen Aerzte daselbst. Unter redaktion der* Herrn Doktoren LICHTENSTADT und SEIDLITZ.—*St. Petersburg.* 8vo. I. Band. 1831; II. Band. 1832.

*Communications respecting the Epidemic Cholera at St. Petersburg, in the Summer of* 1831; *by Medical Practitioners in that City. Arranged and edited by* Drs. LICHTENSTEIN and SEIDLITZ.—*St. Petersburg.* Two Vols. 8vo. 1831, 1832. Pp. 266, 321.

2. *Rapport sur le Cholera Morbus de Moscou.* Par F. C. MARCUS, M.D., Inspecteur et Médecin en Chef de l'Hopital Galitzine, &c. &c. —*Moscou,* 1832. 4to. pp. 450.

*Report on the Cholera Morbus of Moscow.* By F.C. MARCUS, M.D., Chief Physician to the Hospital Galitzin, &c.—*Moscow,* 1832. 4to. pp. 450.

3. *Specimen Historico-Medicum de Choleræ Asiaticæ itinere per Belgium Septentrionale.* Auctore A. C. G. SUERMAN.—*Trajecti ad Rhenum,* 1835. 8vo. pp. 289.

*A Medico-Statistical Account of the Epidemic Cholera in Holland, during the Years* 1832, 1833. By A. C. G. SUERMAN, M.D. &c.—*Utrecht,* 1835. 8vo. pp. 289.

HAVING, in two articles in our last Number, (see Review of Dr. Mackintosh and Mr. Parkin,) had occasion to notice the subject of Cholera, and to lament, in common with all rational observers, how ignorant we still remain both of its pathology and cure, we cannot put aside the works of which we have transcribed the titles, and which have just reached us from our kind friends abroad, without making one or two observations on the most important subject of which they treat. Our readers will hardly expect that we are now about to enter at any length on the subject of epidemic cholera. As, however, we have no good grounds for concluding that that dreadful malady will not visit us again, we are anxious to point out to our readers how desirable it is that they should now, when their minds are calm and comparatively uninfluenced by their feelings, their prejudices, or their theories, endeavour to make themselves acquainted with the actual phenomena of the disease; the circumstances under which it occurred in different countries; its progress and decrease; its real symptoms; the appearances on dissection; its actual and relative mortality under different modes of treatment, on a large scale. It cannot be concealed, that as yet we are utterly ignorant of any mode of preventing or curing cholera, and that the great majority of the works which have been published on this subject, in this and other countries, not only do not afford us any substantial hope of assistance in the event of a return of the disease, but are humiliating proofs of the unphilosophical way in which medicine is generally cultivated, of the fallacy of the grounds on which much of our received pathology rests, and of the lamentable imperfection of our therapeutic means.

With the view of removing some portion of these deficiencies in regard to cholera, we would earnestly recommend the perusal of works of the same class as those the titles of which we have placed at the head of this notice,—namely, of the historical and statistical kind,—which are composed principally of important facts, not of fanciful or absurd theories. We have much yet to learn of the phenomena and habitudes of cholera, before we can hope to attain a consistent pathology of it; and it is the business of rational medicine, in the present state of our knowledge, to occupy itself principally with these. It is thus we can alone hope to augment our means both of prevention and cure; and, if our hopes should still be disappointed, we shall at least feel that we have been proceeding in the only path which sound philosophy acknowledges.

The works before us are all of extraordinary merit; and we hope, on some future occasion, to lay a summary of the more important facts contained in them before our readers. We refer to them now, chiefly to point them out as among the very best that have been published on the subject. They form a striking contrast with the majority of the works on cholera which have appeared in this country. We cannot lay them aside without transferring to our pages one or two of the very important and interesting statistical facts wherewith they are enriched.

In the epidemic of Moscow, in 1831-2, the total number of patients treated for cholera was 8,609; of whom, 4,695 (that is, more than one-half,) died. The proportion of cholera patients to the whole population was about 1 in 36.

Besides observations on every subject relating to this epidemic, the treatise of Dr. Marcus contains fifty cases minutely detailed, (in Latin,) with the appearances on dissection. In the dissections, particular attention was paid to the state of the blood and the other fluids of the body, especially the bile, and urine, and the serous discharges from the bowels. An elaborate memoir on the chemical analysis of these is given by M. Hermann, from which we make the following extract:

| | Proportion of Coagulum and Serum. | | Specific Gravity of the Serum. |
|---|---|---|---|
| | Coagulum. | Serum. | |
| Blood of a young man in good health . . | 43 | 57 | 1027 |
| Blood of a healthy pregnant woman . . | 44.75 | 55.25 | 1023 |
| Blood of a girl attacked with cholera, before the watery evacuations . . . . | 50 | 50 | 1027 |
| Blood drawn from men labouring under cholera, with watery evacuations, and who recovered | *a.* 55 | 45 | 1028 |
| | *b.* 60.5 | 39.7 | 1032 |
| | *c.* 62.5 | 37.5 | 1028 |
| Blood taken from a cholera patient, four hours before death . . . . . . . | 60 | 40 | 1036 |

In all the cases, the clot and serum evinced acid qualities on the application of tincture of litmus, except in the four last, (the individuals actually under cholera with watery discharges,) in which it showed alkaline reaction.

In Holland, the disease, out of a population of 2,427,206, attacked 19,037, in both the epidemics of 1832 and 1833. In the former year, the proportion of cases of disease to the whole population was, *in rural*

*districts*, 1.54 in the 1000; and, *in towns*, 8.93 in the 1000: in the latter year, the proportions were 1.08 and 7.42; giving an average, in both epidemics, of 8.17 in the 1000 for towns, and 1.31 for the country. The proportion of deaths in both epidemics was as follows:

1. Proportion of deaths to the whole population:
   In towns, . . 4.36 to 1000 inhabitants,
   In the country, 0.68 to 1000 ditto.
2. Proportion of deaths to the whole number of cases of cholera:
   In towns, . . 1 in 1.87,
   In the country, 1 in 1.91.

While we are writing this, we learn from a medical friend in Prussia, who had recently made a tour through Austria, Hungary, and Bohemia, that he found cholera prevailing everywhere, and everywhere, as heretofore, committing its ravages, unchecked by all the efforts of medical science.

